# Density Functional Theory Analysis of Deltamethrin and Its Determination in Strawberry by Surface Enhanced Raman Spectroscopy

**DOI:** 10.3390/molecules23061458

**Published:** 2018-06-15

**Authors:** Tao Dong, Lei Lin, Yong He, Pengcheng Nie, Fangfang Qu, Shupei Xiao

**Affiliations:** 1 College of Biosystems Engineering and Food Science, Zhejiang University, Hangzhou 310058, China; 21613052@zju.edu.cn (T.D.); linlei2016@zju.edu.cn (L.L.); yhe@zju.edu.cn (Y.H.); ffqu@zju.edu.cn (F.Q.); 180312@zju.edu.cn (S.X.); 2 Key Laboratory of Spectroscopy Sensing, Ministry of Agriculture, P.R. China; 3 State Key Laboratory of Modern Optical Instrumentation, Zhejiang University, Hangzhou 310058, China

**Keywords:** deltamethrin, strawberry, density functional theory, surface enhanced Raman spectroscopy, gold nanoparticles, PLS, BIPLS

## Abstract

Deltamethrin is widely used in pest prevention and control such as red spiders, aphids, and grubs in strawberry. It is important to accurately monitor whether the deltamethrin residue in strawberry exceeds the standard. In this paper, density functional theory (DFT) was used to theoretically analyze the molecular structure of deltamethrin, gold nanoparticles (AuNPs) and silver nanoparticles (AgNPs) were used to enhance the surface enhanced Raman spectroscopy (SERS) detection signal. As a result, the theoretical Raman peaks of deltamethrin calculated by DFT were basically similar to the measured results, and the enhancing effects based on AuNPs was better than that of AgNPs. Moreover, 554, 736, 776, 964, 1000, 1166, 1206, 1593, 1613, and 1735 cm^−1^ could be determined as deltamethrin characteristic peaks, among which only three Raman peaks (736, 1000, and 1166 cm^−1^) could be used as the deltamethrin characteristic peaks in strawberry when the detection limit reached 0.1 mg/L. In addition, the 500–1800 cm^−1^ SERS of deltamethrin were analyzed by the partial least squares (PLS) and backward interval partial least squares (BIPLS). The prediction accuracy of deltamethrin in strawberry ($R_{p}^{2}$ = 0.93, *RMSE_p_* = 4.66 mg/L, *RPD* = 3.59) was the highest when the original spectra were pretreated by multiplicative scatter correction (MSC) and then modeled by BIPLS. In conclusion, the deltamethrin in strawberry could be qualitatively analyzed and quantitatively determined by SERS based on AuNPs enhancement, which provides a new detection scheme for deltamethrin residue determination in strawberry.

## 1. Introduction

Deltamethrin([(S)-cyano-(3-phenoxyphenyl)methyl](1R,3R)-3-(2,2-dibromoethenyl)-2,2-dimet-hylcyclopropane-1-carboxylate), a benzimidazole derivative which belongs to the absorption of a broad-spectrum fungicide, has been widely used in strawberry red spiders, aphids, and grub disease prevention. Eating strawberries with excess deltamethrin residue could cause diseases such as cancer, blood disease, and immune system disorders [1]. In accordance with the provision of the maximal residue of pesticides in food in China (GB 29705-2013), the deltamethrin in a strawberry cannot exceed 1 mg/kg. Traditional methods for determining deltamethrin pesticides include high-performance liquid chromatography (HPLC) [2], fluorescence quantification [3], ion exchange chromatography [4] and capillary electrophoresis [5]. For example, Mao et al. [6] achieved simultaneous determination of tralomethrin, deltamethrin, and its related compounds by HPLC with radiometric detection. Liu et al. [7] successfully applied immune magnetic nanoparticles separation coupled with surface plasmon resonance sensor to detect deltamethrin. The results revealed that the detection sensitivity for deltamethrin detection line of deltamethrin concentrations could reach from 0.01 mg/L to 1 mg/L. Wang et al. [8] realized the rapid detection of deltamethrin based on AuNPs modified with 2-mercapto-6-nitrobenzothiazole. The detection limit of deltamethrin was 0.005 μg/g by UV–vis spectroscopy. Although these methods are highly sensitive, the measurement process was complicated and the reagent was expensive [9].

Compared with the complex methods mentioned above, surface enhanced Raman spectroscopy (SERS) has attracted wide attention in recent years. The principle of SERS is that the molecular signals in compounds can be enhanced when the surface of detected materials adsorb some nanoscale rough metals (such as gold, silver, and copper) [10]. Beside this, SERS also has the advantages of simple pretreatment, convenient equipment, and fast detection speed, which makes it suitable for the rapid screening of complex compounds [11]. In the aspect of SERS determination of pesticide residue, many scholars have carried out related studies. He et al. [12] detected the pesticide residue in apple using a surface swab capture method followed by SERS, the results indicated that the swab-SERS method was simple, sensitive, rapid (10 min), and could quantitatively enough detect pesticides on raw agricultural produce like pears, carrots, and melons etc. Lin et al. [13] applied the SERS based on silver nanoparticle to analyze the Raman peaks of thiabendazole pesticides. It was indicated that 782, 1012, 1284, 1450, and 1592 cm^−1^ could be selected as the thiabendazole pesticides Raman characteristic peaks. However, there were few studies on the application of Raman spectroscopy (RS) or SERS in the determination of deltamethrin. Guo et al. [14] successfully applied RS to the identification of deltamethrin and acetamiprid. The results showed that Raman spectra at 574 and 736 cm^−1^ can be used to identify deltamethrin. However, the detection line was too high (2500 mg/kg). Perna et al. [15] studied Raman spectroscopy of human neuronal and epidermal cells exposed to an insecticide mixture of chlorpyrifos and deltamethrin. The results suggested that Raman micro spectroscopy could investigate cells from people who were frequently exposed to chemicals such as deltamethrin pesticides using principal component analysis (PCA). Peng et al. [16] achieved the identification and determination of deltamethrin and acetamiprid in apple using SERS. The results showed that detection line of these pesticide residues in apple using SERS could reach 0.005 μg/cm^2^ and 0.002 μg/cm^2^ respectively.

However, there were few reports on qualitative judgment and quantitative detection of deltamethrin in strawberry using SERS. Therefore, the main purpose of this paper is that analyzing the characteristic peaks of deltamethrin by density functional theory (DFT) and qualitatively and quantitatively determine deltamethrin residue in strawberry using SERS combined with chemometric methods. In summary, it was of great significance to provide a method of accurately detecting deltamethrin residue in strawberry. 

## 2. Results and Discussion

### 2.1. Molecular Structure of Deltamethrin Calculated by DFT

**Figure 1:**
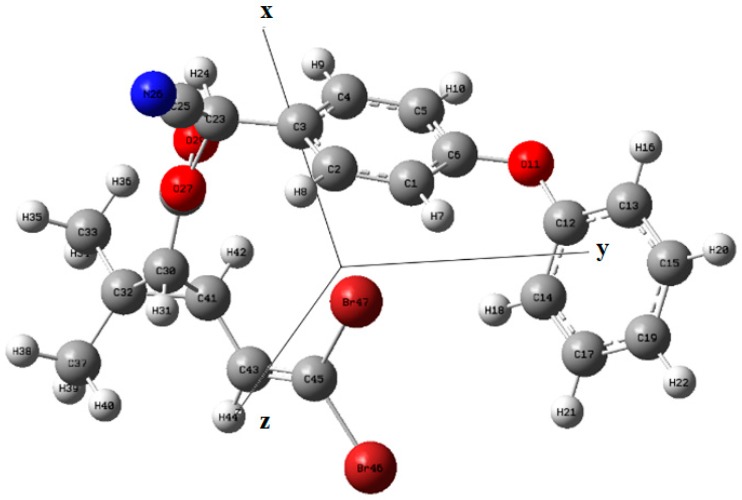
Simulated molecular structure of deltamethrin by DFT.

Deltamethrin (molecular formula: C_22_H_19_Br_2_NO_3_) is consisted of C-Br, C=C, C=N, C=O, C-H, benzene ring and N-H groups. There are several isomers of deltamethrin molecular, for example, (αR)–deltamethrin and trans-deltamethrin [17]. DFT, a common method for molecular geometry optimization and frequency vibration calculation, can describe the ground state physical properties of atoms and molecules [18]. In this paper, DFT was applied to calculate the deltamethrin molecule and optimize its structure in Gaussian.v09 software. Figure 1 shows the atomic coordinates of deltamethrin an isomer with para-substitution in the Cartesian coordinates, reflecting the relative positions of different atoms in the three-dimensional space as well as providing data support for describing the atom position accurately. The deltamethrin molecular calculated by DFT is one of the isomers of deltamethrin, but the spectral vibrational forms in Raman spectra of the isomers are similar [19]. Table S1 as a Supplementary Materials shows the atomic coordinates of deltamethrin. 

Figure 1 and Table S1 directly reflects the detailed information of atoms in deltamethrin, including the relative size of atoms, the types of elements, the form of chemical bonds, the spatial distribution of atoms and the types of connections between atoms. It provided a reliable theoretical basis for the study of the internal and surface vibration forms of deltamethrin molecules.

### 2.2. Deltamethrin SERS and its Assignment of Raman Peaks

**Figure 2:**
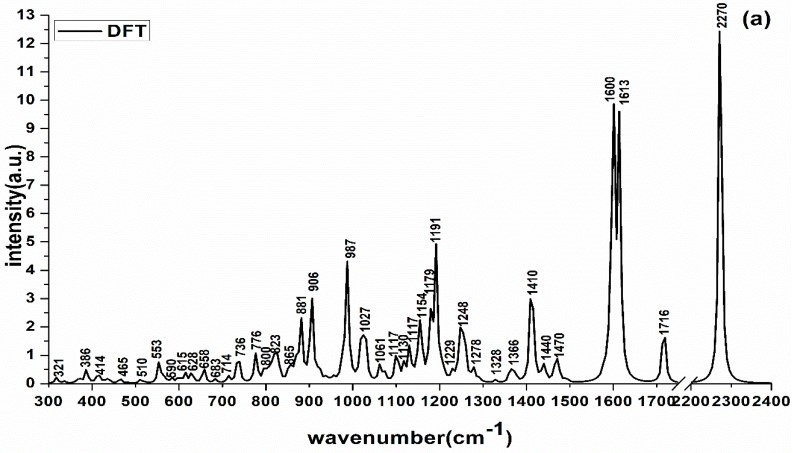

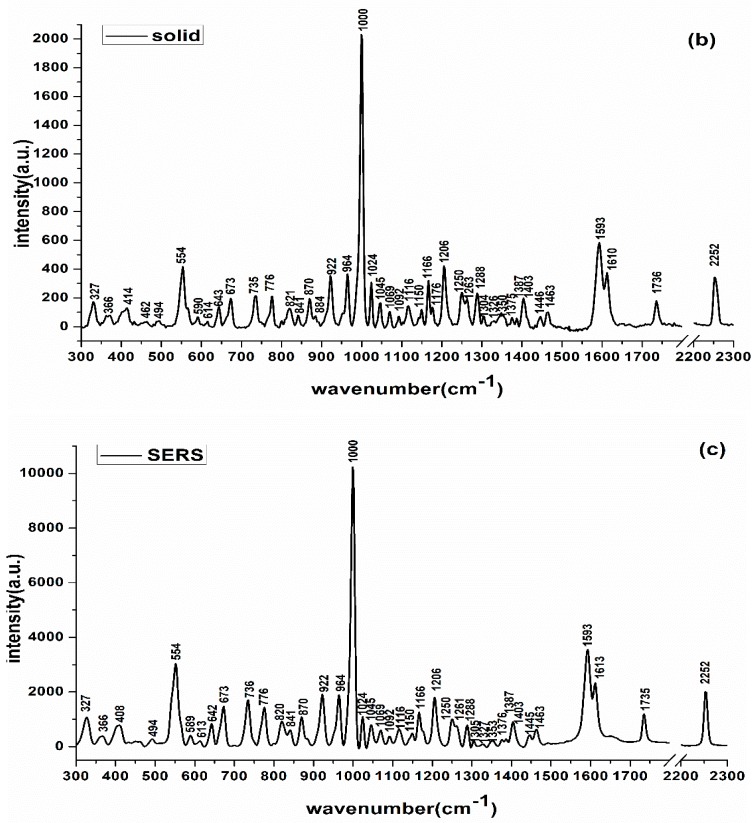
The RS of deltamethrin: (**a**) the theory calculation by density functional theory; (**b**) RS of deltamethrin solid; (**c**) SERS of deltamethrin solution.

The vibrational positions and forms of different chemical bonds in deltamethrin molecule can be obtained by Hartree–Fock wave function [20]. In Gaussian.v09 software, the vibrational form of related chemical bond calculated by Hartree–Fock wave function can be obtained. In order to verify the matching degree between the DFT-calculated Raman peaks and experiment-detected Raman peaks of deltamethrin, the deltamethrin RS calculated by DFT, the RS of solid deltamethrin and the SERS of deltamethrin solution (dissolved in acetonitrile) were compared and analyzed. Figure 2 is the RS of deltamethrin, where Figure 2a is the deltamethrin RS calculated by DFT, Figure 2b is the RS of solid deltamethrin and Figure 2c is the SERS of deltamethrin solution based on gold nanoparticles (AuNPs) (100 mg/L). The assignments of Raman peaks of deltamethrin are listed in Table 1.

**Figure 3:**
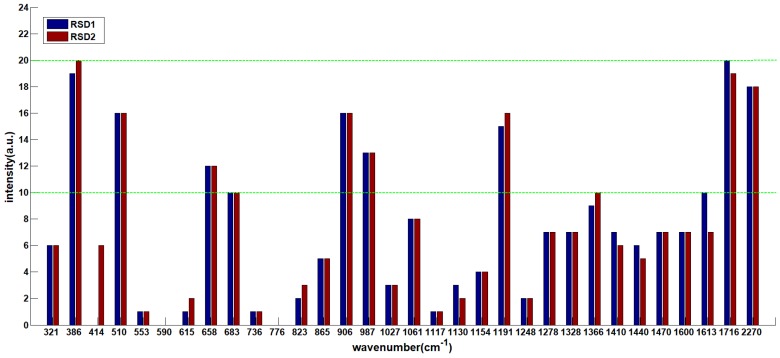
Raman shift deviation between the Raman characteristic peaks of deltamethrin and the Raman characteristic peaks calculated by DFT.

Figure 3 is the Raman shift deviation between the measured Raman characteristic peaks of deltamethrin and the Raman characteristic peaks calculated by DFT, where Raman shift deviation one (RSD1) is the deviation between Raman characteristic peaks of solid deltamethrin and Raman characteristic peaks calculated by DFT, and Raman shift deviation two (RSD2) is the deviation between the Raman characteristic peaks of the SERS of deltamethrin solution and Raman characteristic peaks calculated by DFT.

It can be seen from Figure 2 that the DFT-calculated Raman peaks were basically consistent with the RS of solid deltamethrin and SERS of its solution. Combined with Table 1 and Figure 3, there was no RSD between the Raman characteristic peaks of deltamethrin and the DFT-calculated Raman characteristic peaks at 590 and 776 cm^−1^. Most RSD1 and RSD2 were less than 10 cm^−1^. Although some Raman peaks (386, 510, 658, 906, 987, 1191, 1716, and 2270 cm^−1^) calculated by DFT differed from those of RS and SERS, their Raman shifts (less than 20 cm^−1^) were within a reasonable range, which indicated that the position of Raman peaks detected by SRES were feasible and reliable. As for these weak Raman peaks (841, 964, 1045, 1166, 1350, and 1387 cm^−1^) which appeared in the actual detection of deltamethrin but not in the DFT theoretical calculation, their vibrational forms could be found in the relevant literature [21,22,23,24].

For the SERS of deltamethrin, the Raman peaks of 554, 736, 776, 922, 964, 1000, 1166, 1206, 1593, 1613, 1735, and 2252 cm^−1^ intensity were stronger than others. 554 cm^−1^ was the stretching vibration of C-Br and C-C groups’ inner surface bending and stretching vibration. 736 cm^−1^ was the C-H deformable vibration and outer surface bending, and 776 cm^−1^ belonged to the ring stretching and C=N inner surface bending. 922 cm^−1^ was assigned to the ring stretching and C-H deformable vibration and outer surface bending, 964 cm^−1^ was the C-H deformable vibration and outer surface bending and 1000 cm^−1^ was the C-H inner surface bending and ring stretching vibration. 1166 cm^−1^ was the C-H inner surface bending and ring stretching vibration. 1206 cm^−1^ was C-C groups’ inner surface bending. The 1593 and 1613 cm^−1^ belonged to the C=C inner surface bending and stretching. Moreover, the 1735 cm^−1^ was the C=O stretching and inner surface bending, and 2252 cm^−1^ was the C=N stretching and inner surface bending. The attribution of these peaks could be found on Perna’s and Lin’s research [25,26]. As a result, these peaks could be used as the SERS of deltamethrin.

### 2.3. Comparison of Two Nano Enhancers 

**Figure 4:**
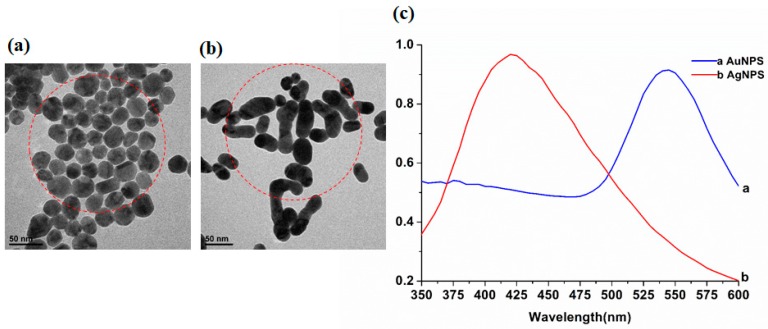
Transmission electron microscopy (TEM) images of: (**a**) gold nanoparticles (AuNPs); (**b**) silver nanoparticles (AgNPs); (**c**) their UV–vis spectra.

Because of the strong interaction with electromagnetic radiation in the visible region, the optical properties of gold and silver make them one of the unique noble metals [25]. Figure 4 shows transmission electron microscopy (TEM) images (Figure 4a–b) and spectra (Figure 4c) of gold nanoparticles (AuNPs) and silver nanoparticles (AgNPs) by UV–vis spectroscopy respectively. Table 2 shows the red selected area (Figure 4) average particle size of AuNPs and AgNPs.

As can be seen in Figure 4 that the UV–vis characteristic absorption peaks of AgNPs and AuNPs were at 423 nm and 543 nm, respectively. Combined with Table 2, in the area of the same size, the number of AuNPs (number: 41) was more than that of AgNPs (number: 24). The minimum size of AuNPs was 16.7 nm, the maximum was 36.7 nm, the average was 27.8 nm, and the standard deviation was 5.6 nm. While the minimum size of AgNPs was 16 nm, the maximum was 70 nm, the average was 36.3 nm, and the standard deviation was 15.6 nm. It was indicated that the particle size of AuNPs was more uniform and the distribution was more concentrated than those of AgNPs.

### 2.4. Deltamethrin Characteristic Peaks

**Figure 5:**
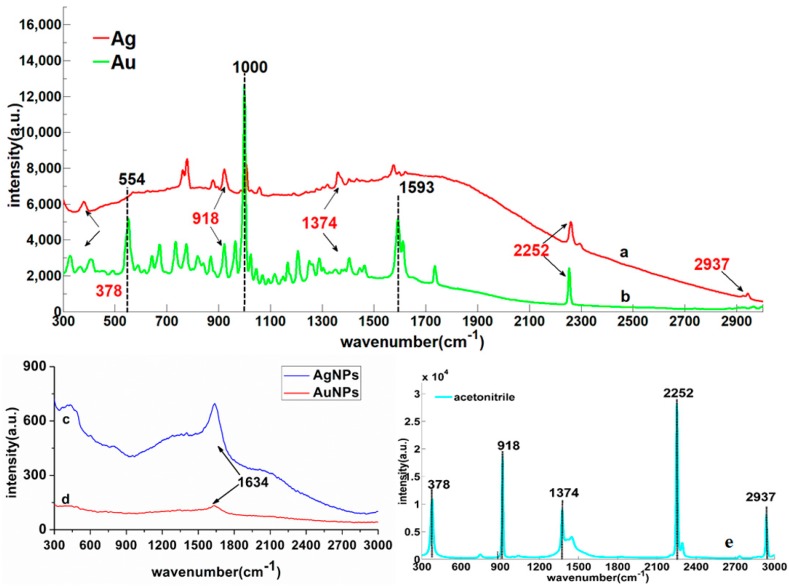
The SERS of deltamethrin solution based on two nanoparticles: (a) AgNPs; (b) AuNPs; the SERS of two nanoparticles; (c) AgNPs; (d) AuNPs; the RS of acetonitrile; (e) acetonitrile.

To investigate the optimal nanoparticles, the enhancement effects of AuNPs and AgNPs were compared and analyzed in this study. The SERS of deltamethrin based on AgNPs and AuNPs are shown in Figure 5a,b. In order to exclude the effects of AgNPs, AuNPs, and acetonitrile Raman signals, the Raman spectra of AgNPs, AuNPs, and acetonitrile were collected, as shown in Figure 5c–e.

As can be seen from Figure 5a,b that the number of deltamethrin characteristic peaks and enhancement effects based on AuNPs on deltamethrin molecule was significantly better than those of AgNPs, especially in 554, 1000, and 1593 cm^−1^. The reason might be that the particle size of AuNPs was more uniform, the distribution was more concentrated, the agglomeration state was closer and the permittivity of surrounding medium was better than those of AgNPs. Thus, the surface plasmon resonance of AuNPs can be stimulated more easily, resulting in the higher detection sensitivity [27]. Beside this, the Raman spectrum of AgNPs and AuNPs only had a faint signal at 1634 cm^−1^ (Figure 5c,d), indicating that AgNPs and AuNPs themselves had no strong Raman characteristic peaks and did not have a negative effect on experimental results. Therefore, the AuNPs was more suitable as the substrate in SERS to detect the deltamethrin residue in strawberry for the following study. In addition, except those Raman peaks (378, 918, 1374, 2252, and 2937 cm^−1^) which belonged to acetonitrile (Figure 5e), 554, 736, 776, 964, 1000, 1166, 1206, 1593, 1613, and 1735 cm^−1^ could be selected as deltamethrin characteristic peaks.

### 2.5. Analysis of 500–1800 cm^−1^ SERS of Deltamethrin Pesticides in Strawberry

In this paper, the SERS of 101 strawberry samples based on AuNPs enhancement were obtained and pretreated by standard normal variation (SNV), multiplicative scatter correction (MSC), de-trending (DT), and first-derivative (1st-Der). Meanwhile, considering that the Raman peaks of deltamethrin were mainly distributed in the range of 500–1800 cm^−1^, the original SERS of deltamethrin in strawberry were preprocessed and then modeled by partial least squares (PLS) [28] and backward interval partial least squares (BIPLS) [29]. 

**Figure 6:**
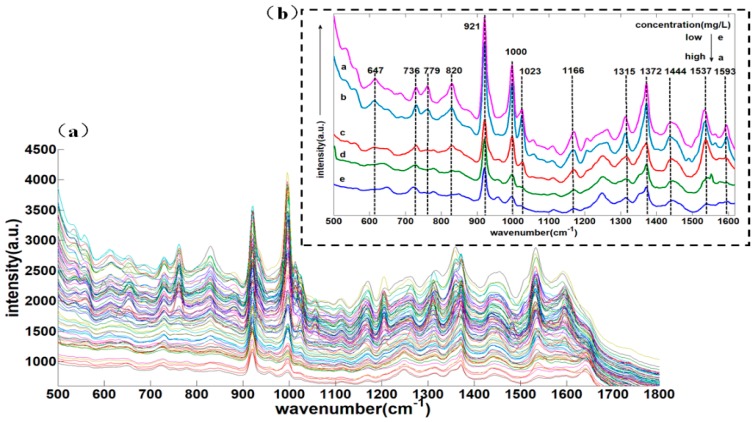
(**a**) 500–1800 cm^−1^ SERS spectra of different concentrations of deltamethrin in strawberry; (**b**) the SERS spectra of five concentrations of deltamethrin in strawberry.

The sample set portioning based on joint x-y distance (SPXY) method [30] was used to divide the samples into two groups, among which 70 samples were calibrated and 31 samples were validated. The SERS of 101 samples are shown in Figure 6 and the prediction performance are presented in Table 3. According to Figure 6a,b, the SERS absorbance intensity decreased gradually with the decrease of deltamethrin content at those characteristic peaks (647, 736, 779, 820, 921, 1000, 1023, 1166, 1315, 1372, 1444, 1537, and 1593 cm^−1^). 921 and 1372 cm^−1^ belonged to the acetonitrile and deltamethrin common characteristic peaks. Combined with Table 2, except those Raman peaks (1315 and 1537 cm^−1^) which did not appear in the SERS of deltamethrin, all the characteristic peaks of deltamethrin in strawberry were basically the same as deltamethrin solution. The reason might be that other substances in the strawberries had strong Raman signal. The concentration of deltamethrin pesticides in strawberry was in the range of 0.1 mg/L to 60 mg/L in this experiment and when the concentration was the lowest, the characteristic peaks signal was weak at 647, 779, 820, 1023, and 1593 cm^−1^ and only 736, 1000, and 1166 cm^−1^ could be identified. Therefore, 736, 1000, and 1166 cm^−1^ could be selected as characteristic peaks of deltamethrin pesticides in strawberry. Meanwhile, the three peaks were clearly visible when deltamethrin was at 0.1 mg/L and the detection limit was lower than the maximal pesticide residue of food (1 mg/L) in China, which indicated that the stoichiometric method could be used to quantitatively analyze whether there was excess deltamethrin residue in strawberry.

**Figure 7:**
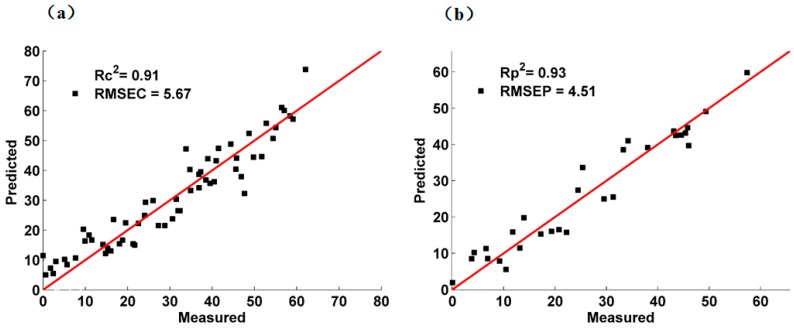
Scatter diagram of calibration set and prediction set by MSC: (**a**) calibration set; (**b**) prediction set.

The PLS and BIPLS prediction effects of deltamethrin in strawberry are presented in Table 3. No matter PLS or BIPLS was used, the $R_{c}^{2}$ and $R_{p}^{2}$ achieved more than 0.88 and the prediction accuracy of deltamethrin in strawberry was the best ($R_{c}^{2}$ = 0.94, *RMSE_c_* = 4.41 mg/L; $R_{p}^{2}$ = 0.93, *RMSE_p_* = 4.66 mg/L, *RPD* = 3.59) when the SERS were processed with MSC and modeled by BIPLS (Figure 7a: calibration set; Figure 7b: prediction set), revealing that the deltamethrin in strawberry could be quantitatively determined by SERS based on AuNPs. 

According to the PLS and BIPLS model performance, on the one hand, the prediction results of BIPLS model were better than those of PLS model. The reason might be that BIPLS could better extract the characteristic variables of SERS, which reduced the amount of sub-intervals of the worst or collinear variables [29]. On the other hand, no matter which spectral preprocess methods were used, the prediction effects of MSC were the optimum both in PLS and BIPLS model. The reason might be that MSC eliminated the effects of uneven sample distribution and filling density, which improved the spectral resolution and separated the main characteristic peaks for quantitative analysis [31]. 

## 3. Materials and Methods 

### 3.1. Experimental Instruments and Reagents

For this study, the experimental instruments mainly included: (1) RmTracer-200-HS portable Raman spectrometer combined with a 785 nm excitation wavelength diode-stabilized stimulator (Opto Trace Technologies, Inc., Mountain View, CA, USA); (2) Agilent 1290 Ultra Performance Liquid Chromatography Combined Photodiode Array Detector (Agilent Technologies, Santa Clara, CA, USA); (3) JW-1024 low-speed centrifuge (Anhui Jia Instrument and Equipment Co., Ltd. Anhui, China); (4) The FEI Tecnai G2 F20 S-TWIN transmission electron microscope (USA FEI Corporation, Hillsboro, OR, USA); (5) Vortex-Genie 2/2T vortex mixer (Shanghai Ling early Environmental Protection Instrument Co., Ltd., Shanghai, China); (6) the column, Agilent ZORBAX SB-C18, 150 × 2.1 mm, 3.5 μm (Agilent Technologies, Santa Clara, CA, USA); (7) ZNCL intelligent thermostat magnetic stirrer (Zhengzhou Ya-Rong Instrument Co., Ltd., Zhengzhou, China).

Moreover, the experimental reagents included: (1) deltamethrin (99.8% purity, Sigma-Aldrich, St. Louis, MO, USA); (2) acetonitrile (chromatographically purity, Amethyst Chemicals); (3) silver nitrate, perchlorate, trisodium citrate, chloroauric acid (ethylenediamine-N-propyls lane); (4) sodium chloride (Analytical Pure, National Standards Information Center); (6) organic filter (0.22 μm, Agilent Technologies, Inc., Santa Clara, CA, USA). 

### 3.2. Experimental Methods

#### 3.2.1. Sample Preparation

In this study, 101 pesticide-free red-cheek strawberry (Japan 99) were selected as the experimental samples. The specific experimental procedure were as follows. First, 101 different concentrations deltamethrin standard solutions (0–100 mg/L, 1 mg/L per gradient) were prepared and sprayed on 101 pesticide-free strawberry, where one strawberry sample was set as a blank contrast. Second, the corresponding strawberry samples were picked after 24 h. Third, the Raman detection samples were prepared as follows. 10 g strawberry sample was added into 50 mL centrifuge tube, and then 10 mL acetonitrile, 3 g sodium chloride, and 4 g sodium acetate were added in turn. After mixing 1 min in the vortex mixer of 400 r/min, the mixed solution was put into centrifuge at the speed of 10,000 r/min for 2 min. Finally, the supernatant was filtered by a 0.22 μm organic membrane. The supernatant were divided into two groups, one group were detected by ultra-high performance liquid chromatography (UHPLC) [32] and the other group were detected by SERS.

#### 3.2.2. Silver Nano-Substrate and Gold Nano-Substrate Preparation

The preparation of silver nanoparticle was established on the basis of the Lee-Meisel trisodium citrate heating reduction method [33]. The silver nano-substrate preparation process was as follows. First, a silver nitrate solution (180 mg/L) was heated quickly for boiling at a 360 °C on a constant temperature magnetic stirrer. Second, 1% trisodium citrate solution was dropwise added and stirred at 200 r/min. Third, the magnetic stirrer was closed after the solution turned to gray-green in 25 min. Fourth, when the heating temperature was dropping, the silver nitrate solution was poured into a centrifuge tube and then a small amount of supernatant was remained after centrifugation. Finally, ultrapure water was mixed with ultrasonic vibration, and the gold colloid was stored in dark at 4 °C after repeating purification.

The trisodium citrate heating reduction method was slightly modified according to the literature for the preparation of gold nanoparticle [34]. The gold nanoparticle preparation process was as follows. First, a chloroauric acid solution (50 mg/L) was heated for boiling at 360 °C on a constant temperature magnetic stirrer, and then 4 mL of trisodium citrate solution (5 mg/mL) was added. Second, the mixture was stirred at 100 r/min until the gold sol changed into the color of the wine red. Third, when the solution cooled, the gold gel solution was poured into a centrifuge tube and was stored in dark at 4°C after repeating purification.

### 3.3. Raman Spectrum Acquisition

Before Raman spectrum acquisition, the instrument should be calibrated using a 785 nm excitation wavelength before data acquisition. The parameters were set as follows: a power of 200 mw, a scanning range of 200 to 3300 cm^−1^, an optical resolution of 2 cm^−1^, an integration time of 10 s, and an average spectral value of three times. The solid deltamethrin RS collection was that deltamethrin powder was in quartz plate with glass slides flattened and the spectra were acquired with matching microscope platform. When collecting the SERS of samples, 500 μL silver colloid, 100 μL test solution, and 100 μL sodium chloride were added in turn into a 2 mL quartz bottle, and then it was placed at a liquid sample pool.

### 3.4. Density Functional Theory (DFT)

The principle of DFT is the exchange-correlation functionals. The complexity of various exchange-correlation functionals is different. It is generally divided into five levels with increasing accuracy: local density approximation (LDA), generalized gradient approximation (GGA), meta generalized gradient approximation (mGGA), hybrid, and double hybrid functional [35]. Parr et al. [36] gave sharp definitions for chemical concepts in various branches of chemistry. Besides, the performance of Becke three-parameter Lee–Yang–Parr functional (B3LYP) functional in combination with various basis sets has been extensively tested for molecular geometries, vibrational frequencies, ionization energies and electron affinities, dipole and quadrupole moments, atomic charges, infrared intensities, and magnetic properties [37]. Among the various of functions and basis sets in DFT, the hybrid functional B3LYP with the 6-31G (d,p) basis set has been commonly used in the Raman spectroscopic calculation of biological molecules [38]. In this paper, B3LYP/6-31G (d,p) was used for the theoretical simulation and calculation of deltamethrin molecules.

### 3.5. Spectral Preprocessing Methods

The noise caused by the equipment and the interference of the fluorescence background in the Raman signal could affect the detection results. Therefore, five pretreatment methods were applied to preprocessing the original Raman spectra in this paper [39]. For this paper, four spectral preprocessing methods were applied to dealing with the original spectra. The principle of standard normal variation (SNV) [40] algorithm is that the absorbance values of each wavelength point satisfies a certain distribution in each spectra, and the spectral correction was performed according to this assumption. The basic idea of multiplicative scatter correction (MSC) [31] algorithm is to use an ideal spectra to represent all the samples, and the original spectra is corrected with the slope and intercept of the linear equation. The idea of the de-trending (DT) algorithm is that the spectral absorbance and wavelength are first fitted into a trend line d according to the polynomial; then, the trend line d is subtracted from the original spectra x to achieve the effect of this trend [41]. First-derivative (1st- Der) [42] can eliminate interference from other backgrounds and distinguish the overlapping peaks, which improves spectral resolution, sensitivity and the signal to noise ratio of the spectra to a certain extent. In this paper, all data analysis were based on MATLAB R2014a (The Math-Works, Natick, MA, USA), Gaussian.v09 (Gaussian, Inc., Wallingford, CT, USA), and OMNIC v8.2 (Infrared spectrum processing software, Thermo Fisher Scientific, Waltham, MA, USA).

### 3.6. Modeling Method

Partial least squares (PLS) is a most widely-used regression modeling method in spectral data analysis for its flexibility and reliability in dealing with the redundant spectral data. When PLS is applied to dealing with the spectral data, the spectral matrix is decomposed first and the main principal component variables are obtained, then the contribution rate of each principal component is calculated [28]. The flexibility of PLS makes it possible to establish a regression model in the case where the number of samples is less than the number of variables.

Backward interval partial least squares(BIPLS)is a variable selection method mainly used to filter the wavelength range of PLS model and reduce the amount of sub-intervals of the worst or collinear variables [29]. The algorithm steps are mainly as follows: (a) Divide the whole spectrum into k bands of equal width. (b) Leave a section from the k section spectrum. Carry out the PLS regression on the remaining (k-1) section and establish the sub model of the quality to be measured. Set aside each paragraph in turn to get the k sub model. (c) Measure the accuracy of each model by RMSECV value. Delete the reserved segment corresponding to the highest precision sub model, and take the sub model as the first base model. (d) Leave one more section in the remaining (k-1) section of the spectrum and use the remaining (k-2) segments to model the PLS. Each section is set aside in order to obtain the (k-1) sub model to remove the reserved segments corresponding to the sub model of the minimum RMSECV value. Take the sub model as the second base model. Repeat the process until there is one remaining wave band. (e) Investigate the RMSEP value of each base model according to steps b to d. Select the best and minimum RMSECV among all the base models, and the corresponding interval combination is the best combination.

### 3.7. Model Evaluation Index

In this experiment, the modeling effect is evaluated by the coefficient of determination (*R*^2^), the root mean square error (*RMSE*), and the residual predictive deviation (*RPD*). The coefficient of determination *R*^2^ reflects the level of intimacy between variables, the *RMSE* reflects the accuracy of the model, and *RPD* reflects the predictive ability of the model. The higher the *RPD*, the lower the RMSE, and the closer the *R*^2^ is to 1, the better the performance of the prediction model. In this paper, $R_{c}^{2}$ and $R_{p}^{2}$ represent the coefficient of the determination of the calibration set and the prediction set respectively, while *RMSE_c_* and *RMSE_p_* represent the root mean square error of the calibration set and the prediction set, respectively. In addition, the *RPD* was suggested to be at least three for agriculture applications; while 2 < *RPD* < 3 indicated a model with a good predictive ability; 1.4 < *RPD* < 2 was an intermediate model needing some improvement; and *RPD* < 1.4 indicated that the model had a poor predictive ability [42].

## 4. Conclusions

In this paper, DFT was used to simulate and optimize the molecular structure of deltamethrin and SERS technology was initially applied to detect deltamethrin pesticides in strawberry. The conclusions were as follows: (1) 554, 736, 776, 964, 1000, 1206, 1166, 1593, 1613, and 1735 cm^−1^ can be selected as deltamethrin characteristic peaks and three Raman peaks (736, 1000, and 1166 cm^−1^) could be determined as the characteristic peaks of deltamethrin in strawberry to qualitatively judge whether there was excess the standard (1 mg/L) of deltamethrin residue in strawberry; (2) the deltamethrin in strawberry could be quantitatively detected using SERS based on AuNPs enhancement, and the prediction results ($R_{p}^{2}$ = 0.93) was the best combined with MSC pretreatment and BIPLS model. In conclusion, the deltamethrin in strawberry could be qualitative estimated and quantitatively detected by SERS based on AuNPs, which provides a new detection scheme for pesticide residues.

## Figures and Tables

**Table 1:** The assignment of Raman peaks of deltamethrin.

Calculation (cm^−1^)	Raman of Solid (cm^−1^)	SERS of Deltamethrin (cm^−1^)	Assignment
321	327(w)	327(w)	υ(C-C)ip
386	367(w)	366(vw)	δ(C-C-C)ip
414	414(w)	408(w)	υ_ring_
465	462(vw)	-	υ_ring_
510	494(vw)	494(vs)	υ(C-Br)ip
553	554(m)	554(s)	υ(C-Br)ip + υ(C-C)ip
590	590(vw)	589(w)	υ_ring_
615	614(vw)	613(vs)	υ_ring_
628	-	-	υ_ring_
658	643(w)	643(w)	υ_ring_+υ(C-C-C)ip
683	673(w)	673(w)	υ_breathe_
714	-	-	υ_breathe_
736	735(w)	736(m)	δ(C-H)oop
776	776(w)	776(m)	υ_ring_+δ(C=N)ip
800	800(vs)		υ_breathe_
823	821(w)	820(w)	υ_breathe_
-	841(w)	841(w)	υ_breathe_
865	870(w)	870(w)	δ(C-H)oop
881	884(w)	-	δ(C-H)oop
906	922(m)	922(m)	υ_ring+_δ(C-H)oop
-	964(m)	964(m)	δ(C-H)oop
987	1000(vs)	1000(vs)	υ_ring+_δ(C-C)ip
1027	1024(m)	1024(w)	υ_ring_
-	1045(w)	1045(w)	υ(C-C)ip
1061	1069(w)	1069(w)	δ(C-H)ip
-	1092(w)	1092(w)	δ(C-H)ip
1117	1116(w)	1116(w)	δ(C-H)opp
1130	-	-	δ(C-H)ip
1154	1150	1150	υ_ring_
-	1166(w)	1166(w)	υ_ring_+δ(C-H)ip
1179	1176(w)	-	υ_ring_
1191	1206(m)	1206(m)	υ(C-C)ip
1229	-	-	υ_ring_
1248	1250(w)	1250(w)	υ_ring_+δ(C-H)ip
1278	1289(w)	1288(w)	δ(C-H)opp
-	1304(w)	1305(vs)	δ(C-H)ip
1328	1326(w)	1327(vs)	δ(C-H)opp
-	1350(w)	1353(vs)	δ(C-H) ip
1366	1375(vw)	1376(vw)	δ(C-H)opp
-	1387(vw)	1387(vs)	δ(C-H) opp
1410	1403(w)	1404(w)	δ(C-H)opp
1440	1446(w)	1445(vs)	δ(C-H) opp
1470	1463(w)	1463(w)	δ(C-H)ip
1600	1593(s)	1593(s)	υ(C=C)ip
1613	1610(m)	1613(m)	υ(C=C)ip
1716	1736(w)	1735(w)	υ(C=O)ip
2270	2252(m)	2252(m)	υ(C=N)ip

Note: vs = very strong; s = strong; m = medium; w = weak; υ = stretching; opp = outer surface bending; ip = Inner surface bending; δ = deformable vibration.

**Table 2:** Average particle size of AuNPs and AgNPs.

Types	Number	Min	Max	Average Value	Standard Deviation
AuNPs	41	16.7	36.7	27.8	5.6
AgNPs	24	16	70	36.3	15.6

**Table 3:** Results of pre-processing method for calibration and prediction model.

Methods	Pre-Processing Method	Calibration Set		Prediction Set		
		$R_{c}^{2}$	RMSEC (mg/L)	$R_{p}^{2}$	RMSEP (mg/L)	RPD
PLS	Original	0.89	5.84	0.89	6.48	2.91
	MSC	0.90	5.54	0.90	6.21	3.01
	SNV	0.90	5.68	0.88	6.12	2.92
	DT	0.90	4.93	0.88	6.39	2.95
	1st-Der	0.88	5.87	0.88	6.66	2.82
BIPLS	Original	0.89	5.65	0.92	5.07	3.30
	MSC	0.94	4.41	0.93	4.66	3.59
	SNV	0.91	5.67	0.93	4.51	3.78
	DT	0.90	5.37	0.91	5.17	3.41
	1st-Der	0.90	5.79	0.91	5.26	3.30

## Supplementary Materials

The following are available online.

## References

[1] Rodríguez R., Picó Y., Font G., Mañes J. (2002). Analysis of deltamethrin and procymidone in fruits and vegetables by capillary electrophoresis-electrospray mass spectrometry. J. Chromatogr..

[2] Rong T., Yang R., And J.C.Y., Zhu H. (2003). Polyphenolic profiles in eight apple cultivars using high-performance liquid chromatography (hplc). J. Agric. Food Chem..

[3] Yi L. (2009). A highly sensitive fluorescence probe for fast thiol-quantification assay of glutathione reductase. Angew. Chem..

[4] Hugli T.E., Moore S. (1972). Determination of the tryptophan content of proteins by ion exchange chromatography of alkaline hydrolysates. J. Biol. Chem..

[5] Kasicka V. (2006). Recent developments in capillary electrophoresis and capillary electrochromatography of peptides. Electrophoresis.

[6] Mao J., Erstfeld K.M., Fackler P.H. (1993). Simultaneous determination of tralomethrin, deltamethrin, and related compounds by hplc with radiometric detection. J. Agric. Food Chem..

[7] Liu X., Li L., Liu Y.Q., Shi X.B., Li W.J., Yang Y., Mao L.G. (2014). Ultrasensitive detection of deltamethrin by immune magnetic nanoparticles separation coupled with surface plasmon resonance sensor. Biosens. Bioelectron..

[8] Wang Z.Q., Huang Y., Wang D., Sun L., Dong C., Fang L., Zhang Y.J., Wu A.G. (2018). A rapid colorimetric method for the detection of deltamethrin based on gold nanoparticles modified with 2-mercapto-6-nitrobenzothiazole. Anal. Methods.

[9] Önal A. (2007). A review: Current analytical methods for the determination of biogenic amines in foods. Food Chem..

[10] Farquharson S., Gift A., Shende C., Inscore F., Ordway B., Farquharson C., Murren J. (2008). Surface-enhanced raman spectral measurements of 5-fluorouracil in saliva. Molecules.

[11] Doering W.E., Nie S. (2002). Single-Molecule and Single-Nanoparticle SERS: Examining the Roles of Surface Active Sites and Chemical Enhancement. J. Phys. Chem. B.

[12] He L., Chen T., Labuza T.P. (2014). Recovery and quantitative detection of deltamethrin on apples using a surface swab capture method followed by surface-enhanced raman spectroscopy. Food Chem..

[13] Lin L., Wu R., Liu M., Wang X., Yan L. (2015). Surface-enhanced raman spectroscopy analysis of thiabendazole pesticide. Spectrosc. Spectr. Anal..

[14] Guo L.H., Peng Y.K., Li Y.Y., Sagar D., Tao F.F. (2014). Research on identification of deltamethrin and acetamiprid pesticides using raman spectroscopy. J. Food Saf. Qual..

[15] Perna G., Lasalvia M., Capozzi V. (2014). Raman spectroscopy of human neuronal and epidermal cells exposed to an insecticide mixture of chlorpyrifos and deltamethrin. Appl. Spectrosc..

[16] Peng Y., Dhakal S., Chao K., Qin J. (2015). Research on identification and determination of mixed pesticides in apples using surface enhanced Raman spectroscopy. SPIE Sens. Technol. Appl..

[17] Hill B.D., Johnson D.L. (1987). Persistence of deltamethrin and its isomers on pasture forage and litter. J. Agric. Food Chem..

[18] Chermette H. (2015). Chemical reactivity indexes in density functional theory. J. Comput. Chem..

[19] Matysik J., Hildebrandt P., Smit K., Mark F., Gärtner W., Braslavsky S.E., Schaffner K., Schrader B. (1997). Raman spectroscopic analysis of isomers of biliverdin dimethyl ester. J. Pharm. Biomed. Anal..

[20] Montoya A., Truong T.N., Sarofim A.F. (2000). Spin contamination in hartree−fock and density functional theory wavefunctions in modeling of adsorption on graphite. J. Phys. Chem. A.

[21] Altunbek M., Kuku G., Culha M. (2016). Gold nanoparticles in single-cell analysis for surface enhanced raman scattering. Molecules.

[22] Notingher I. (2007). Raman spectroscopy cell-based biosensors. Sensors.

[23] Huang Y.S., Karashima T., Yamamoto M., Hamaguchi H.O. (2003). Molecular-level pursuit of yeast mitosis by time- and space-resolved raman spectroscopy. J. Raman Spectrosc..

[24] Movasaghi Z., Rehman S., Rehman I.U. (2007). Raman spectroscopy of biological tissues. Appl. Spectrosc. Rev..

[25] Perna G., Lasalvia M., D’Antonio P., Quartucci G., Capozzi V. (2011). Characterization of human cells exposed to deltamethrin by means of raman microspectroscopy and atomic force microscopy. Vib. Spectrosc..

[26] Lin L. (2014). Qualitative and Quantitative Analysis of Pesticide Residues in Tea by Surface Enhanced Raman Spectroscopy (SERS).

[27] Kawamura K., Tsujimoto Y., Rabenarivo M., Asai H., Andriamananjara A., Rakotoson T. (2017). Vis-NIR Spectroscopy and PLS Regression with Waveband Selection for Estimating the Total C and N of Paddy Soils in Madagascar. Remote Sens..

[28] Blanchard P., Brüning E. (2015). Mathematical Methods in Physics.

[29] Zou X., Zhao J., Li Y. (2007). Selection of the efficient wavelength regions in ft-nir spectroscopy for determination of ssc of ‘fuji’ apple based on biPLS and fipls models. Vib. Spectrosc..

[30] Xiao S., He Y., Dong T., Nie P. (2018). Spectral analysis and sensitive waveband determination based on nitrogen detection of different soil types using near infrared sensors. Sensors.

[31] Yamaguchi T., Takamura H., Matoba T., Terao J. (1998). Hplc method for evaluation of the free radical-scavenging activity of foods by using 1,1-diphenyl-2-picrylhydrazyl. J. Agric. Chem. Soc. Jpn..

[32] Ding L., Fang Y. (2007). An investigation of the surface-enhanced raman scattering (sers) effect from laser irradiation of ag nanoparticles prepared by trisodium citrate reduction method. Appl. Surf. Sci..

[33] Su Q., Ma X., Dong J., Jiang C., Qian W. (2011). A reproducible sers substrate based on electrostatically assisted aptes-functionalized surface-assembly of gold nanostars. ACS Appl. Mater. Interfaces.

[34] Yang Y., Weaver M.N., Merz K. (2009). Assessment of the “6-31+g** + lanl2dz” mixed basis set coupled with density functional theory methods and the effective core potential: Prediction of heats of formation and ionization potentials for first-row-transition-metal complexes. J. Phys. Chem. A.

[35] Cao Y., Hughes T., Giesen D., Halls M.D., Goldberg A., Vadicherla T.R., Sastry M., Patel B., Sherman W., Weisman A.L. (2016). Highly efficient implementation of pseudospectral time-dependent density-functional theory for the calculation of excitation energies of large molecules. J. Comput. Chem..

[36] Ipatov A., Cordova F., Doriol L.J., Casida M.E. (2009). Excited-state spin-contamination in time-dependent density-functional theory for molecules with open-shell ground states. J. Mol. Struct. Theochem..

[37] Pellizzeri S., Delaney S.P., Korter T.M., Zubieta J. (2013). Using solid-state density functional theory and terahertz spectroscopy to spectroscopically distinguish the various hydrohalide salts of 5-(4-pyridyl) tetrazole. J. Mol. Struct..

[38] Nie P., Dong T., He Y., Qu F. (2017). Detection of soil nitrogen using near infrared sensors based on soil pretreatment and algorithms. Sensors.

[39] Nie P., Dong T., He Y., Xiao S., Qu F., Lin L. (2018). The effects of drying temperature on nitrogen concentration detection in calcium soil studied by nir spectroscopy. Appl. Sci..

[40] Kong W., Zhang C., Huang W., Liu F., He Y. (2018). Application of hyperspectral imaging to detectsclerotinia sclerotiorumon oilseed rape stems:. Sensors.

[41] Dhanoa M.S., Barnes R.J., Lister S.J. (1989). Standard normal variate transformation and de-trending of near-infrared diffuse reflectance spectra. Appl. Spectrosc..

[42] Lin L., Dong T., Nie P., Qu F., He Y., Chu B. (2018). Rapid determination of deltamethrin pesticides in rape by surface enhanced raman spectroscopy. Sensors.

