# Supplementary material for: Density Functional Theory Analysis of Deltamethrin and Its Determination in Strawberry by Surface Enhanced Raman Spectroscopy
Source: Molecules. 2018 Jun 15;23(6):1458. doi: 10.3390/molecules23061458 (PMC6100570; doi:10.3390/molecules23061458)
Supplement: Supplementary file 1 [file molecules-23-01458-s001.pdf]

molecules

MDPI

Density Functional Theory Analysis of Deltamethrin and Its Determination in Strawberry by Surface Enhanced Raman Spectroscopy

Tao Dong [1,2,†], Lei Lin [1,2,†], Yong He [1,2], Pengcheng Nie [1,2,3,*], Fangfang Qu [1,2], and Shupei Xiao [1,2]

[1] College of Biosystems Engineering and Food Science, Zhejiang University, Hangzhou 310058, China; 21613052@zju.edu.cn (T.D.); linlei2016@zju.edu.cn (L.L.); yhe@zju.edu.cn (Y.H.); ffqu@zju.edu.cn (F.Q.); 180312@zju.edu.cn (S.X.)
[2] Key Laboratory of Spectroscopy Sensing, Ministry of Agriculture, Beijing 100125, China
[3] State Key Laboratory of Modern Optical Instrumentation, Zhejiang University, Hangzhou 310058, China
* Correspondence: npc2012@zju.edu.cn; Tel.: +86-571-8898-2456
† These authors contributed equally to this work.

Supplementary materials:

Table S1. Atomic Coordinates.

Z-matrix:							
Charge = 0 Multiplicity = 1							
**Symbol**	**X**	**Y**	**Z**	**Symbol**	**X**	**Y**	**Z**
C1	2.24606	0.58596	0.03708	H24	5.87938	1.80692	-1.03196
C2	3.64122	0.58596	0.03708	C25	5.87842	0.45083	0.79159
C3	4.33876	1.79372	0.03708	N26	5.87817	-0.5491	1.3527
C4	3.64111	3.00222	0.03588	O27	7.30876	1.79393	0.03879
C5	2.24628	3.00215	0.0354	C28	8.73876	1.79403	0.03962
C6	1.54868	1.79394	0.03639	O29	9.39342	2.72693	-0.41581
H7	1.6963	-0.36635	0.03753	C30	9.4027	0.54574	0.65001
H8	4.19073	-0.36655	0.03839	H31	9.11507	0.45473	1.6766
H9	4.19131	3.95437	0.03582	C32	10.93387	0.68085	0.55594
H10	1.69616	3.95443	0.03444	C33	11.38798	1.92863	1.33596
O11	0.11868	1.79418	0.03616	H34	12.29198	1.70929	1.86472
C12	-1.31132	1.79442	0.03593	H35	11.56098	2.73329	0.65222
C13	-2.00875	1.79648	-1.17188	H36	10.62651	2.21028	2.03291
C14	-2.00908	1.79299	1.24438	C37	11.34713	0.82018	-0.92101
C15	-3.40391	1.79591	-1.172	H38	11.46597	1.85591	-1.16195
H16	-1.45916	1.79723	-2.12435	H39	12.27265	0.30801	-1.08225
C17	-3.4039	1.7929	1.24418	H40	10.58991	0.39343	-1.54504
H18	-1.45896	1.79143	2.19657	C41	11.59767	-0.57115	1.15882
C19	-4.1014	1.79396	0.03591	H42	11.30828	-0.66954	2.18423
H20	-3.95359	1.79708	-2.12437	C43	13.12891	-0.43423	1.06845
H21	-3.95411	1.79178	2.19641	H44	13.56153	0.45588	0.6617
H22	-5.201	1.7937	0.036	C45	13.93035	-1.43723	1.50233
C23	5.87876	1.79383	0.03796	Br46	15.74814	-1.02553	1.08491
				Br47	13.73962	-1.6321	3.39276

Table 1 shows the atomic coordinates of deltamethrin in the Cartesian coordinates, reflecting the relative positions of different atoms in the three-dimensional space as well as providing data support for describing the atom position accurately. Table 1 directly reflects the detailed information of atoms in deltamethrin, including the relative size of atoms, the types of elements, the form of chemical bonds, the spatial distribution of atoms and the types of connections between atoms. It provided a reliable theoretical basis for the study of the internal and surface vibration forms of deltamethrin molecules. For example, C1 stand for No. 1 carbon atom and in Cartesian coordinate system, the X, Y and Z coordinates of C1 are 2.24606, 0.58596 and 0.03708 respectively.

```
# Raman Activity Spectrum
# X-Axis:   Frequency (cm-1)
# Y-Axis:   Intensity

# Peak information
#                X                    Y
#        16.1326000000          0.3219000000
#        22.2764000000          1.2721000000
#        27.6849000000          0.4053000000
#        33.6664000000          1.0073000000
#        42.6720000000          8.2125000000
#        43.9703000000          2.1579000000
#        56.4516000000          4.2565000000
#        70.1417000000          2.0286000000
#        73.0643000000          0.6322000000
#        90.1638000000          1.5232000000
#        97.6414000000          0.9791000000
#       100.7419000000          4.6907000000
#       126.1932000000          2.5970000000
#       137.7371000000          1.2448000000
#       147.8663000000          1.2624000000
#       161.4655000000          1.9173000000
#       177.6148000000          1.3355000000
#       198.5618000000          0.6041000000
#       213.3210000000          1.2886000000
#       232.0404000000          1.0943000000
#       233.8567000000          3.8811000000
#       250.2096000000          1.4160000000
#       255.7828000000          7.3497000000
#       273.4590000000          0.4224000000
#       279.2456000000          2.5429000000
```

#	318.4249000000	2.7700000000
#	336.4080000000	0.9485000000
#	360.3800000000	0.3739000000
#	370.3101000000	2.6424000000
#	387.2323000000	6.9262000000
#	403.7310000000	0.1125000000
#	410.6746000000	2.4031000000
#	417.7103000000	2.9751000000
#	433.5361000000	2.1514000000
#	440.8613000000	0.9161000000
#	464.3768000000	2.4135000000
#	482.7417000000	0.3496000000
#	510.5279000000	1.5877000000
#	519.4306000000	0.8489000000
#	554.8318000000	11.7211000000
#	567.6270000000	2.6193000000
#	583.9508000000	2.3107000000
#	599.5712000000	3.0995000000
#	614.4355000000	5.0853000000
#	629.8812000000	5.6745000000
#	657.1966000000	8.1406000000
#	683.2058000000	1.5663000000
#	685.1131000000	0.5325000000
#	713.5092000000	3.5039000000
#	734.7334000000	6.3462000000
#	737.4578000000	10.2177000000
#	776.2495000000	6.9611000000
#	777.7598000000	8.7434000000
#	796.6820000000	6.2023000000
#	805.3973000000	6.4188000000
#	816.7727000000	11.4492000000
#	822.7269000000	8.4366000000
#	828.4054000000	10.9279000000
#	854.4803000000	9.8414000000
#	867.9794000000	11.1661000000
#	880.9831000000	34.2786000000
#	897.6304000000	1.4231000000
#	905.5071000000	48.4524000000
#	922.6308000000	7.4434000000
#	938.6996000000	0.4738000000
#	939.7514000000	1.8824000000
#	947.9763000000	0.5280000000

#	955.6670000000	2.1440000000
#	969.4598000000	0.1364000000
#	975.3662000000	7.1599000000
#	986.7526000000	61.1625000000
#	989.7980000000	7.6235000000
#	998.4977000000	0.6624000000
#	1011.6881000000	1.2333000000
#	1021.0930000000	28.9856000000
#	1029.3042000000	21.8561000000
#	1062.7157000000	9.2820000000
#	1072.8926000000	5.1431000000
#	1101.2364000000	17.8042000000
#	1104.8024000000	1.0846000000
#	1117.9126000000	9.0498000000
#	1131.0713000000	19.8198000000
#	1147.1069000000	5.5658000000
#	1154.5405000000	28.7496000000
#	1159.5769000000	8.7428000000
#	1178.8490000000	34.4533000000
#	1191.4872000000	76.8864000000
#	1206.5827000000	8.4077000000
#	1230.2038000000	5.3939000000
#	1246.1040000000	13.9023000000
#	1250.8523000000	27.5044000000
#	1258.3970000000	19.2363000000
#	1277.4664000000	8.3481000000
#	1289.1836000000	2.5720000000
#	1299.3609000000	0.2950000000
#	1328.3608000000	1.7113000000
#	1360.7316000000	2.9151000000
#	1364.1670000000	5.7556000000
#	1371.3022000000	4.0278000000
#	1376.0778000000	2.2064000000
#	1411.8288000000	75.4523000000
#	1420.9608000000	2.0974000000
#	1437.9764000000	8.8052000000
#	1442.1136000000	2.3330000000
#	1444.0137000000	2.4231000000
#	1460.8655000000	1.1290000000
#	1468.5403000000	17.9203000000
#	1477.1876000000	3.2642000000
#	1492.4210000000	2.3538000000

#	1579.2578000000	6.6397000000
#	1593.8026000000	31.0054000000
#	1598.8359000000	45.8709000000
#	1601.2944000000	120.8415000000
#	1613.7979000000	156.4968000000
#	1716.2036000000	43.4169000000
#	2272.8336000000	328.1207000000
#	2971.1050000000	109.5558000000
#	2976.7072000000	416.2880000000
#	3005.7412000000	71.1299000000
#	3042.6176000000	54.2660000000
#	3045.7759000000	176.1142000000
#	3055.3578000000	60.5372000000
#	3058.8436000000	57.1778000000
#	3088.3844000000	34.5979000000
#	3108.4850000000	123.5493000000
#	3118.9160000000	30.9650000000
#	3123.3286000000	41.3363000000
#	3125.6664000000	31.2711000000
#	3126.8489000000	196.4964000000
#	3134.5397000000	43.3619000000
#	3138.0179000000	89.7730000000
#	3143.6667000000	142.9651000000
#	3148.3563000000	386.8748000000
#	3149.7073000000	137.7110000000
#	3151.4086000000	134.8360000000

Spectra

#	X	Y	DY/DX
	200.0000000000	0.0669731220	-0.0053720527
	206.2000000000	0.0526430358	0.0035272385
	212.4000000000	0.1217397859	0.0110983102
	218.6000000000	0.0763508148	-0.0051697492
	224.8000000000	0.0956510466	0.0120310739
	231.0000000000	0.3020630943	0.0566719177
	237.2000000000	0.2470126035	-0.0465871978
	243.4000000000	0.1412218400	0.0044217739
	249.6000000000	0.2994209240	0.0428305363
	255.8000000000	0.6148312725	-0.0102160276
	262.0000000000	0.1982656795	-0.0375669450
	268.2000000000	0.1002688992	-0.0020380059
	274.4000000000	0.1432769836	0.0128294134
	280.6000000000	0.2031335061	-0.0288775146
	286.8000000000	0.0633925145	-0.0095031760
	293.0000000000	0.0336197833	-0.0021205495
	299.2000000000	0.0273419766	-0.0001107888
	305.4000000000	0.0326175440	0.0020811787
	311.6000000000	0.0659887418	0.0114118915
	317.8000000000	0.2150871357	0.0155917533
	324.0000000000	0.0867883812	-0.0156480060
	330.2000000000	0.0524022602	0.0015767465
	336.4000000000	0.0918060947	-0.0006763079
	342.6000000000	0.0403681100	-0.0045894198
	348.8000000000	0.0297838047	0.0001263438
	355.0000000000	0.0404663121	0.0039332607
	361.2000000000	0.0785764768	0.0039203455
	367.4000000000	0.1624692783	0.0307773361
	373.6000000000	0.1676042243	-0.0232538548
	379.8000000000	0.1548332324	0.0187803449
	386.0000000000	0.4900971906	0.0645376088
	392.2000000000	0.2255087781	-0.0480599114
	398.4000000000	0.0964060859	-0.0052492721
	404.6000000000	0.1147431125	0.0112580602
	410.8000000000	0.2573757231	0.0078721437
	417.0000000000	0.2855753145	0.0072247903
	423.2000000000	0.1265731157	-0.0168237359
	429.4000000000	0.1234366681	0.0156386006
	435.6000000000	0.1727364891	-0.0206723479
	441.8000000000	0.1128222057	-0.0131080541

448.0000000000	0.0483110449	-0.0040594242
454.2000000000	0.0442764772	0.0025065443
460.4000000000	0.1031137871	0.0213743700
466.6000000000	0.1466684310	-0.0283932243
472.8000000000	0.0467268275	-0.0057000171
479.0000000000	0.0364693180	0.0018637715
485.2000000000	0.0363006230	-0.0043983027
491.4000000000	0.0225100740	-0.0005014839
497.6000000000	0.0252813939	0.0013348454
503.8000000000	0.0462274866	0.0069950793
510.0000000000	0.1345124228	0.0091263779
516.2000000000	0.0901189910	0.0005348283
522.4000000000	0.0701853445	-0.0102830561
528.6000000000	0.0416435445	-0.0007960981
534.8000000000	0.0474723339	0.0025853346
541.0000000000	0.0787939817	0.0086700585
547.2000000000	0.1960013349	0.0374664271
553.4000000000	0.7602571577	0.1187503736
559.6000000000	0.3928377706	-0.0765804690
565.8000000000	0.2682730827	0.0143267212
572.0000000000	0.1559769200	-0.0227191549
578.2000000000	0.1175058097	0.0076436128
584.4000000000	0.2127381662	-0.0086976504
590.6000000000	0.1129884141	-0.0028028714
596.8000000000	0.1998552526	0.0340690087
603.0000000000	0.1927873543	-0.0252225761
609.2000000000	0.1936714361	0.0269037971
615.4000000000	0.3921995328	-0.0362909321
621.6000000000	0.1825332869	-0.0037189260
627.8000000000	0.3633169099	0.0599882849
634.0000000000	0.2334466113	-0.0481770252
640.2000000000	0.1001250383	-0.0060981312
646.4000000000	0.1061563653	0.0082890312
652.6000000000	0.2700841752	0.0591089106
658.8000000000	0.5094743126	-0.0841411644
665.0000000000	0.1418011238	-0.0233793891
671.2000000000	0.0739557352	-0.0036268371
677.4000000000	0.0822253444	0.0078731313
683.6000000000	0.1719524926	-0.0003895596
689.8000000000	0.0772397236	-0.0098679135
696.0000000000	0.0527515670	-0.0005087406
702.2000000000	0.0631897756	0.0042873966

708.4000000000	0.1323656799	0.0234129260
714.6000000000	0.2777858689	-0.0250363428
720.8000000000	0.1436617503	-0.0028982686
727.0000000000	0.2195170022	0.0310812683
733.2000000000	0.7347677861	0.1438638350
739.4000000000	0.7779622601	-0.1553235931
745.6000000000	0.2257070554	-0.0339392362
751.8000000000	0.1221177397	-0.0066486807
758.0000000000	0.1089153135	0.0016807107
764.2000000000	0.1481051871	0.0126980290
770.4000000000	0.3384891150	0.0637489061
776.6000000000	1.0731384633	0.0548599640
782.8000000000	0.4435891126	-0.0775028867
789.0000000000	0.2732931433	0.0064391758
795.2000000000	0.5420323428	0.0680590672
801.4000000000	0.5237299090	0.0177014948
807.6000000000	0.6078826796	-0.0489982417
813.8000000000	0.7808858782	0.1243439326
820.0000000000	1.0687776079	-0.0017874408
826.2000000000	1.0705453106	0.0172664297
832.4000000000	0.5760317183	-0.1092876345
838.6000000000	0.2613742634	-0.0165673021
844.8000000000	0.2506110989	0.0128060466
851.0000000000	0.5274425963	0.0960267562
857.2000000000	0.6645483339	-0.0849743142
863.4000000000	0.6063830751	0.0711370604
869.6000000000	0.9986970971	-0.0750416449
875.8000000000	1.1084640471	0.1746613123
882.0000000000	2.3158309200	-0.2535209954
888.2000000000	0.7738729703	-0.0951723472
894.4000000000	0.6626448009	0.0458966392
900.6000000000	1.4743118713	0.2879199671
906.8000000000	3.0111324233	-0.4241087098
913.0000000000	0.8561896116	-0.1361427531
919.2000000000	0.6009249522	0.0349982873
925.4000000000	0.5251092859	-0.0874305849
931.6000000000	0.2486325317	-0.0133267109
937.8000000000	0.2807510182	0.0183347976
944.0000000000	0.2226498804	-0.0117027712
950.2000000000	0.2176841477	0.0046720659
956.4000000000	0.2955220961	-0.0082320340
962.6000000000	0.2482040466	0.0069460709

968.8000000000	0.3988086701	0.0478763490
975.0000000000	0.9600339220	0.0864241939
981.2000000000	1.6394835828	0.3019453003
987.4000000000	4.3119579736	-0.2272588816
993.6000000000	1.3781397849	-0.2766558021
999.8000000000	0.5743415927	-0.0571670447
1006.0000000000	0.4171691978	0.0025451505
1012.2000000000	0.6019166069	0.0521718651
1018.4000000000	1.5590997764	0.3126405102
1024.6000000000	1.7181743512	-0.1184687214
1030.8000000000	1.5638378962	-0.2491303380
1037.0000000000	0.4787617768	-0.0734911039
1043.2000000000	0.2426960156	-0.0171697650
1049.4000000000	0.1930870897	-0.0003113760
1055.6000000000	0.2596161714	0.0283750059
1061.8000000000	0.6823242043	0.0651441632
1068.0000000000	0.4241528466	-0.0196808747
1074.2000000000	0.4411857948	-0.0517448047
1080.4000000000	0.2006418619	-0.0139327629
1086.6000000000	0.1846711961	0.0063788409
1092.8000000000	0.3066555407	0.0407776660
1099.0000000000	0.9778601837	0.1894798336
1105.2000000000	0.7723617464	-0.1343527843
1111.4000000000	0.4381560933	0.0108397904
1117.6000000000	0.8070798120	0.0294242162
1123.8000000000	0.5958156467	0.0195936866
1130.0000000000	1.3571396711	0.1414247842
1136.2000000000	0.7063466423	-0.0988095581
1142.4000000000	0.5838534492	0.0441828920
1148.6000000000	1.0940158606	0.0860398295
1154.8000000000	2.2465545205	-0.0130464149
1161.0000000000	1.2313033835	-0.1754145754
1167.2000000000	0.6831242170	-0.0047774829
1173.4000000000	1.1308139346	0.1865969645
1179.6000000000	2.6443192182	-0.1184256936
1185.8000000000	2.1854728096	0.2526948385
1192.0000000000	4.9266955587	-0.3147538753
1198.2000000000	1.4790943581	-0.2624100290
1204.4000000000	0.9223171534	0.0219569728
1210.6000000000	0.5587212043	-0.0835617510
1216.8000000000	0.3027317635	-0.0155417204
1223.0000000000	0.2893717287	0.0126793818

1229.2000000000	0.5287227537	0.0431307906
1235.4000000000	0.4402347355	0.0002407634
1241.6000000000	0.7955589389	0.1399827471
1247.8000000000	1.9855416546	0.1471029468
1254.0000000000	1.7946763795	-0.1541828975
1260.2000000000	1.3577925937	-0.2342582321
1266.4000000000	0.4650237311	-0.0530567509
1272.6000000000	0.4010016811	0.0328204150
1278.8000000000	0.5899744566	-0.0720248183
1285.0000000000	0.2688993838	-0.0081740744
1291.2000000000	0.2301725699	-0.0315743382
1297.4000000000	0.1169173577	-0.0061785134
1303.6000000000	0.0785053187	-0.0050320049
1309.8000000000	0.0612119924	-0.0013983374
1316.0000000000	0.0573215715	0.0005898609
1322.2000000000	0.0743965041	0.0066396178
1328.4000000000	0.1459152957	-0.0002890324
1334.6000000000	0.0768051080	-0.0059359068
1340.8000000000	0.0633278019	0.0005162095
1347.0000000000	0.0771943809	0.0042581963
1353.2000000000	0.1281140214	0.0161564039
1359.4000000000	0.3658573700	0.0630858199
1365.6000000000	0.5112863019	-0.0423576170
1371.8000000000	0.4437211324	-0.0152752563
1378.0000000000	0.2766195010	-0.0351798217
1384.2000000000	0.1683112937	-0.0030120152
1390.4000000000	0.1937965422	0.0107443268
1396.6000000000	0.3203892435	0.0342555549
1402.8000000000	0.7585499314	0.1344257489
1409.0000000000	2.9817101302	0.6951904116
1415.2000000000	2.6537056243	-0.6241334409
1421.4000000000	0.8289619925	-0.1189439631
1427.6000000000	0.3999184006	-0.0252608849
1433.8000000000	0.4623105822	0.0561172304
1440.0000000000	0.7065504811	-0.0475428519
1446.2000000000	0.3852835413	-0.0585455415
1452.4000000000	0.2131753080	-0.0051696843
1458.6000000000	0.2897883933	0.0313825509
1464.8000000000	0.6693532299	0.1296356417
1471.0000000000	0.8719012205	-0.1570717173
1477.2000000000	0.4292175955	-0.0349517538
1483.4000000000	0.1930155095	-0.0177127971

1489.6000000000	0.1875632560	0.0151672769
1495.8000000000	0.1522314887	-0.0217787805
1502.0000000000	0.0837340174	-0.0045335956
1508.2000000000	0.0682471296	-0.0012218943
1514.4000000000	0.0644308650	-0.0001496941
1520.6000000000	0.0654859290	0.0004553768
1526.8000000000	0.0695757755	0.0009636958
1533.0000000000	0.0770478119	0.0015110858
1539.2000000000	0.0883872649	0.0022082323
1545.4000000000	0.1049529456	0.0032018648
1551.6000000000	0.1292377772	0.0047601381
1557.8000000000	0.1678232638	0.0074977353
1564.0000000000	0.2292581978	0.0130763676
1570.2000000000	0.3491884183	0.0288155889
1576.4000000000	0.6869286419	0.0933025168
1582.6000000000	1.0046850525	0.0332426463
1588.8000000000	1.9357632248	0.3239832247
1595.0000000000	5.2806449391	0.5908170891
1601.2000000000	9.8652234052	-0.3049522434
1607.4000000000	5.0918358934	-0.0212882898
1613.6000000000	9.6045769799	0.0908922826
1619.8000000000	3.1245234589	-0.6618329568
1626.0000000000	1.1085648670	-0.1445087334
1632.2000000000	0.5677790909	-0.0510124496
1638.4000000000	0.3502454270	-0.0237514809
1644.6000000000	0.2409008351	-0.0129610750
1650.8000000000	0.1783265767	-0.0077857912
1657.0000000000	0.1395810347	-0.0049389251
1663.2000000000	0.1147950841	-0.0031787917
1669.4000000000	0.0991074869	-0.0019370510
1675.6000000000	0.0903882658	-0.0008838545
1681.8000000000	0.0883625415	0.0002785884
1688.0000000000	0.0950346385	0.0020392367
1694.2000000000	0.1173812372	0.0056931731
1700.4000000000	0.1787104660	0.0161462648
1706.6000000000	0.3814580444	0.0613748997
1712.8000000000	1.3994755018	0.3378228915
1719.0000000000	1.6131252860	-0.3732633015
1725.2000000000	0.4129027441	-0.0728064679
1731.4000000000	0.1736917656	-0.0191812859
1737.6000000000	0.0984522885	-0.0074940236
1743.8000000000	0.0654025534	-0.0036974829

1750.0000000000	0.0479963164	-0.0021201012
1756.2000000000	0.0364492135	-0.0013346478
1762.4000000000	0.0239088826	-0.0008408622
1768.6000000000	0.0195192968	-0.0005949546
1774.8000000000	0.0109642208	-0.0003724927
1781.0000000000	0.0089739798	-0.0002759385
1787.2000000000	0.0074797945	-0.0002100424
1793.4000000000	0.0063296566	-0.0001635493
1799.6000000000	0.0054255798	-0.0001298168
1805.8000000000	0.0047021107	-0.0001047533
1812.0000000000	0.0041141849	-0.0000857449
1818.2000000000	0.0036299581	-0.0000710689
1824.4000000000	0.0032264127	-0.0000595585
1830.6000000000	0.0028865783	-0.0000504046
1836.8000000000	0.0025977210	-0.0000430339
1843.0000000000	0.0023501354	-0.0000370326
1849.2000000000	0.0021363198	-0.0000320969
1855.4000000000	0.0019504024	-0.0000280006
1861.6000000000	0.0017877337	-0.0000245726
1867.8000000000	0.0016445939	-0.0000216819
1874.0000000000	0.0015179783	-0.0000192274
1880.2000000000	0.0000000000	0.0000000000
1886.4000000000	0.0000000000	0.0000000000
1892.6000000000	0.0000000000	0.0000000000
1898.8000000000	0.0000000000	0.0000000000
1905.0000000000	0.0000000000	0.0000000000
1911.2000000000	0.0000000000	0.0000000000
1917.4000000000	0.0000000000	0.0000000000
1923.6000000000	0.0000000000	0.0000000000
1929.8000000000	0.0000000000	0.0000000000
1936.0000000000	0.0000000000	0.0000000000
1942.2000000000	0.0000000000	0.0000000000
1948.4000000000	0.0000000000	0.0000000000
1954.6000000000	0.0000000000	0.0000000000
1960.8000000000	0.0000000000	0.0000000000
1967.0000000000	0.0000000000	0.0000000000
1973.2000000000	0.0000000000	0.0000000000
1979.4000000000	0.0000000000	0.0000000000
1985.6000000000	0.0000000000	0.0000000000
1991.8000000000	0.0000000000	0.0000000000
1998.0000000000	0.0000000000	0.0000000000
2004.2000000000	0.0000000000	0.0000000000

2010.4000000000	0.0000000000	0.0000000000
2016.6000000000	0.0000000000	0.0000000000
2022.8000000000	0.0000000000	0.0000000000
2029.0000000000	0.0000000000	0.0000000000
2035.2000000000	0.0000000000	0.0000000000
2041.4000000000	0.0000000000	0.0000000000
2047.6000000000	0.0000000000	0.0000000000
2053.8000000000	0.0000000000	0.0000000000
2060.0000000000	0.0000000000	0.0000000000
2066.2000000000	0.0000000000	0.0000000000
2072.4000000000	0.0000000000	0.0000000000
2078.6000000000	0.0000000000	0.0000000000
2084.8000000000	0.0000000000	0.0000000000
2091.0000000000	0.0000000000	0.0000000000
2097.2000000000	0.0000000000	0.0000000000
2103.4000000000	0.0000000000	0.0000000000
2109.6000000000	0.0000000000	0.0000000000
2115.8000000000	0.0101459427	0.0001291362
2122.0000000000	0.0109965831	0.0001457083
2128.2000000000	0.0119588339	0.0001652409
2134.4000000000	0.0130531042	0.0001884256
2140.6000000000	0.0143046890	0.0002161570
2146.8000000000	0.0157452427	0.0002496064
2153.0000000000	0.0174147980	0.0002903262
2159.2000000000	0.0193645738	0.0003404030
2165.4000000000	0.0216609448	0.0004026852
2171.6000000000	0.0243911570	0.0004811275
2177.8000000000	0.0276717227	0.0005813267
2184.0000000000	0.0316610344	0.0007113743
2190.2000000000	0.0365788027	0.0008832555
2196.4000000000	0.0427368835	0.0011152205
2202.6000000000	0.0505898053	0.0014359578
2208.8000000000	0.0608207894	0.0018922684
2215.0000000000	0.0744948519	0.0025639141
2221.2000000000	0.0933460322	0.0035941389
2227.4000000000	0.1203515466	0.0052571603
2233.6000000000	0.1609726082	0.0081214363
2239.8000000000	0.2261131663	0.0134920620
2246.0000000000	0.3401391528	0.0248006415
2252.2000000000	0.5667443392	0.0529444096
2258.4000000000	1.1160257736	0.1436130266
2264.6000000000	2.9878297182	0.5871812384

2270.8000000000	12.4335811127	2.5114741809
2277.0000000000	7.5049481675	-1.8746797043
2283.2000000000	2.0277998715	-0.3405248935
2289.4000000000	0.8619745827	-0.0983303950
2295.6000000000	0.4685617257	-0.0399299443
2301.8000000000	0.2927969717	-0.0198380237
2308.0000000000	0.1998575807	-0.0112212150
2314.2000000000	0.1449512329	-0.0069432416
2320.4000000000	0.1098748630	-0.0045874115
2326.6000000000	0.0861271891	-0.0031861207
2332.8000000000	0.0693130876	-0.0023014905
2339.0000000000	0.0569770506	-0.0017159639
2345.2000000000	0.0476607075	-0.0013131933
2351.4000000000	0.0404539845	-0.0010271413
2357.6000000000	0.0347652858	-0.0008184385
2363.8000000000	0.0301966201	-0.0006626259
2370.0000000000	0.0264723004	-0.0005439640
2376.2000000000	0.0233964859	-0.0004520135
2382.4000000000	0.0208269679	-0.0003796647
2388.6000000000	0.0186584705	-0.0003219625
2394.8000000000	0.0168117143	-0.0002753816
2401.0000000000	0.0152260795	-0.0002373674
2407.2000000000	0.0138545749	-0.0002060382
2413.4000000000	0.0126603204	-0.0001799872
2419.6000000000	0.0116140455	-0.0001581483
2425.8000000000	0.0106922798	-0.0001397035
2432.0000000000	0.0098760275	-0.0001240186
2438.2000000000	0.0000000000	0.0000000000
2444.4000000000	0.0000000000	0.0000000000
2450.6000000000	0.0000000000	0.0000000000
2456.8000000000	0.0000000000	0.0000000000
2463.0000000000	0.0000000000	0.0000000000
2469.2000000000	0.0000000000	0.0000000000
2475.4000000000	0.0000000000	0.0000000000
2481.6000000000	0.0000000000	0.0000000000
2487.8000000000	0.0000000000	0.0000000000
2494.0000000000	0.0000000000	0.0000000000
2500.2000000000	0.0000000000	0.0000000000
2506.4000000000	0.0000000000	0.0000000000
2512.6000000000	0.0000000000	0.0000000000
2518.8000000000	0.0000000000	0.0000000000
2525.0000000000	0.0000000000	0.0000000000

2531.2000000000	0.0000000000	0.0000000000
2537.4000000000	0.0000000000	0.0000000000
2543.6000000000	0.0000000000	0.0000000000
2549.8000000000	0.0000000000	0.0000000000
2556.0000000000	0.0000000000	0.0000000000
2562.2000000000	0.0000000000	0.0000000000
2568.4000000000	0.0000000000	0.0000000000
2574.6000000000	0.0000000000	0.0000000000
2580.8000000000	0.0000000000	0.0000000000
2587.0000000000	0.0000000000	0.0000000000
2593.2000000000	0.0000000000	0.0000000000
2599.4000000000	0.0000000000	0.0000000000
2605.6000000000	0.0000000000	0.0000000000
2611.8000000000	0.0000000000	0.0000000000
2618.0000000000	0.0000000000	0.0000000000
2624.2000000000	0.0000000000	0.0000000000
2630.4000000000	0.0000000000	0.0000000000
2636.6000000000	0.0000000000	0.0000000000
2642.8000000000	0.0000000000	0.0000000000
2649.0000000000	0.0000000000	0.0000000000
2655.2000000000	0.0000000000	0.0000000000
2661.4000000000	0.0000000000	0.0000000000
2667.6000000000	0.0000000000	0.0000000000
2673.8000000000	0.0000000000	0.0000000000
2680.0000000000	0.0000000000	0.0000000000
2686.2000000000	0.0000000000	0.0000000000
2692.4000000000	0.0000000000	0.0000000000
2698.6000000000	0.0000000000	0.0000000000
2704.8000000000	0.0000000000	0.0000000000
2711.0000000000	0.0000000000	0.0000000000
2717.2000000000	0.0000000000	0.0000000000
2723.4000000000	0.0000000000	0.0000000000
2729.6000000000	0.0000000000	0.0000000000
2735.8000000000	0.0000000000	0.0000000000
2742.0000000000	0.0000000000	0.0000000000
2748.2000000000	0.0000000000	0.0000000000
2754.4000000000	0.0000000000	0.0000000000
2760.6000000000	0.0000000000	0.0000000000
2766.8000000000	0.0000000000	0.0000000000
2773.0000000000	0.0000000000	0.0000000000
2779.2000000000	0.0000000000	0.0000000000
2785.4000000000	0.0000000000	0.0000000000

2791.6000000000	0.0000000000	0.0000000000
2797.8000000000	0.0000000000	0.0000000000
2804.0000000000	0.0000000000	0.0000000000
2810.2000000000	0.0000000000	0.0000000000
2816.4000000000	0.0029366820	0.0000379396
2822.6000000000	0.0144163431	0.0001885260
2828.8000000000	0.0156603291	0.0002134472
2835.0000000000	0.0170725649	0.0002429619
2841.2000000000	0.0186848188	0.0002781790
2847.4000000000	0.0223409980	0.0003433202
2853.6000000000	0.0246324729	0.0003976420
2859.8000000000	0.0272967612	0.0004640926
2866.0000000000	0.0304196202	0.0005462647
2872.2000000000	0.0341130356	0.0006491113
2878.4000000000	0.0385254974	0.0007795871
2884.6000000000	0.0452265869	0.0009649822
2890.8000000000	0.0564834522	0.0012470310
2897.0000000000	0.0666297812	0.0015711124
2903.2000000000	0.0790778603	0.0020079755
2909.4000000000	0.0932409731	0.0025948804
2915.6000000000	0.1117882910	0.0034423784
2921.8000000000	0.1367971943	0.0047156892
2928.0000000000	0.1717639494	0.0067250520
2934.2000000000	0.2238686334	0.0101117954
2940.4000000000	0.3035990216	0.0162658091
2946.6000000000	0.4383694039	0.0288167567
2952.8000000000	0.6987706358	0.0590141331
2959.0000000000	1.2958437289	0.1530443751
2965.2000000000	3.2464915240	0.6020158237
2971.4000000000	10.5024899717	1.2951270611
2977.6000000000	17.2119873216	-1.9534386593
2983.8000000000	4.5881330026	-0.9079269344
2990.0000000000	1.8473058324	-0.1869235126
2996.2000000000	1.3314581938	0.0043745180
3002.4000000000	2.2855033369	0.3808877971
3008.6000000000	2.3751202311	-0.4533664969
3014.8000000000	0.9393106152	-0.0830982141
3021.0000000000	0.7166464464	-0.0048836661
3027.2000000000	0.8226158089	0.0415768806
3033.4000000000	1.3713031888	0.1630247351
3039.6000000000	3.8703814625	0.8190173325
3045.8000000000	9.0037697042	-0.2500255777

3052.0000000000	4.5392756972	-0.0507269161
3058.2000000000	4.7845218092	-0.3085965174
3064.4000000000	1.7972871847	-0.2840944460
3070.6000000000	0.9320014819	-0.0575708186
3076.8000000000	0.7864053770	0.0052799359
3083.0000000000	1.0867066543	0.1187446347
3089.2000000000	1.9878081048	-0.1011649600
3095.4000000000	1.3066402819	0.0035835760
3101.6000000000	2.0523400482	0.2846688452
3107.8000000000	5.8060850232	0.4724199198
3114.0000000000	3.8524387854	-0.0827090927
3120.2000000000	6.0075534811	0.6263168072
3126.4000000000	11.9450266243	0.2032776986
3132.6000000000	7.9007968435	0.1035689330
3138.8000000000	10.7643487300	0.4458323793
3145.0000000000	18.9485550327	2.0025081712
3151.2000000000	21.5838105489	-3.2774278221
3157.4000000000	6.0030806663	-1.1358463148
3163.6000000000	2.3629464136	-0.2823204855
3169.8000000000	1.2671214532	-0.1076302695
3176.0000000000	0.7986471272	-0.0523887386
3182.2000000000	0.5536941009	-0.0295687087
3188.4000000000	0.4086678914	-0.0184089651
3194.6000000000	0.3152513760	-0.0122857520
3200.8000000000	0.2513032858	-0.0086342795
3207.0000000000	0.1999379719	-0.0062470134
3213.2000000000	0.1662846006	-0.0047071777
3219.4000000000	0.1377966082	-0.0036051123
3225.6000000000	0.1179288702	-0.0028443353
3231.8000000000	0.1021129118	-0.0022850678
3238.0000000000	0.0893090681	-0.0018644009
3244.2000000000	0.0787927990	-0.0015416923
3250.4000000000	0.0692253168	-0.0012797330
3256.6000000000	0.0619292380	-0.0010813191
3262.8000000000	0.0557361852	-0.0009221103
3269.0000000000	0.0474622865	-0.0007558449
3275.2000000000	0.0431028150	-0.0006537404
3281.4000000000	0.0385947396	-0.0005603663
3287.6000000000	0.0289717608	-0.0004119454
3293.8000000000	0.0265772528	-0.0003619164
3300.0000000000	0.0213890321	-0.0002819354
